# Successful management for vulvar epithelioid sarcoma during pregnancy: a rare case report

**DOI:** 10.3389/fonc.2025.1570151

**Published:** 2025-07-18

**Authors:** Xiaoqiao Guo, Runjun Li, Yan Liu, Ribo Xiong

**Affiliations:** ^1^ Department of Gynecology and Obstetrics, The Third Affiliated Hospital of Southern Medical University, Guangzhou, China; ^2^ Department of Rehabilitation, The Seventh Affiliated Hospital of Southern Medical University, Foshan, China

**Keywords:** pregnancy, epithelioid sarcoma, vulva, case report, cesarean

## Abstract

Vulvar epithelioid sarcoma (ES) in pregnancy is an exceedingly rare condition, and only three reports are available to date. Optimal management is not well established. Herein, we report a 35-year-old woman who presented with a tender mass on the left labia majora at 33 weeks and 4 days of gestation. The patient underwent radical local resection of the lesion in the left vulva and left inguinal lymphadenectomy at 36 weeks and 4 days of gestation. Simultaneously, preterm cesarean section was performed because of mature fetus indicated by ultrasonography. Pathology of the lesion demonstrated a 7×5×5 cm-sized ES with the infiltration of surrounding adipose tissue, and no necrosis, hemorrhage, or venous invasion were identified. Six weeks after surgery, the patient underwent tumor resection due to recurrence. Then, adjuvant radiotherapy was performed with a dose of 40 Gy/10 cycles, followed by 15 Gy/3 cycles with a 10×10 cm field. The patient survived with neither recurrence nor complications at a 12-month follow-up. This case highlights management of a rare disease in pregnancy, with radical local resection of the lesion and lymphadenectomy combined with cesarean section.

## Introduction

The combination of cancer and pregnancy is rare, as already demonstrated by the occurrence of 0.05% to 0.1% in all pregnancies (1). In particular, vulvar epithelioid sarcoma (ES) is an exceedingly rare condition and comprises approximately 1% of soft tissue sarcomas (2). Standard treatment of this rare tumor is radical local excision with unilateral or bilateral lymph node dissection or sentinel node procedure according to guidelines published in 2019 (3). However, recommendations of the previous guidelines were usually not very specific for vulvar ES during pregnancy. To the best of our knowledge, only three vulvar ES cases during pregnancy have been reported in English literature to date (4–6). Two cases involved women who either died after delivery or were lost to follow-up at 16 weeks of gestation. Successful management was achieved in a 36-year-old Japanese woman who underwent tumor resection at 23 weeks of gestation. Then, cesarean section, radical vulvectomy, and lymphadenectomy were performed at 29 weeks of gestation. This is the second successful management reported in a pregnant woman and the first to describe a successful regimen consisting of cesarean section and surgical resection simultaneously in late pregnancy, which is more challenging.

## Case report

### Initial discovery of the mass

A 35-year-old Chinese woman, gravida 4 para 3, had presented to another hospital with a tender nodule on the left labia majora at 33 weeks and 4 days of gestation in 2024. She had noticed a soybean-sized mass on the left side of her vulva 7 months earlier, but its dimensions had been stable until the last 6 weeks (at 27 weeks and 5 days of gestation), when it began to grow. No evidence of comorbidity was noted. No previous gynecologic problems and chronic diseases such as hypertension, diabetes, and obesity were reported. She had no personal or family history of cancer.

### Initial medical check-up

**Figure 1:**
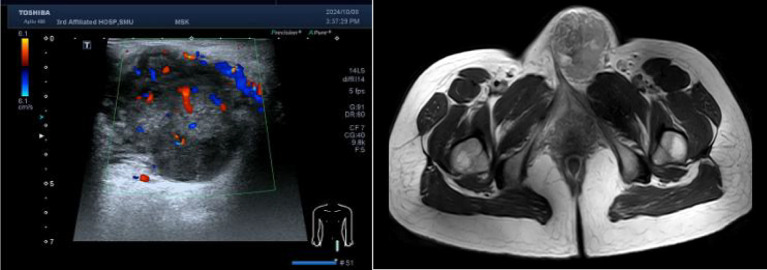
Left: Real-time ultrasonogram of the lesion. Right: Magnetic resonance imaging of the pelvic.

She had visited our hospital with a presumed diagnosis of Bartholin’s gland cyst at 34 weeks and 2 days of gestation. Upon gynecological examination, there was an indurateds and tender vulvar mass measuring 6×7×5 cm arising in the left labia majora. Ultrasonographic examination revealed an irregular solid mass with heterogenous hypoechoic structure, sized 58×49×46 mm (**Figure 1**). The patient underwent needle biopsy for the lesion at 34 weeks and 4 days of gestation, and pathology showed a malignant epithelioid tumor. Five days after needle biopsy, magnetic resonance imaging showed a soft tissue neoplasm measuring 63×59×53 mm over the left labia majora with high signal on T1/T2-weighted imaging (WI). There was a restriction in the diffusion-weighted imaging (DWI) (**Figure 1**). In addition, a computerized tomographic scan of the pelvis, abdomen, and chest suggested no metastasis. At 36 weeks of gestation, Doppler ultrasonography revealed fetal biparietal diameter (BPD), femur length (FL), head circumference (HC), and abdominal circumference (AC) to be 9.0, 7.1, 32.7, and 32.4 cm, respectively. Placental maturity: Grade I; amniotic fluid index (AFI): 14.5 cm; umbilical artery blood flow: S/D = 2.12.

### Surgical treatment

**Figure 2:**
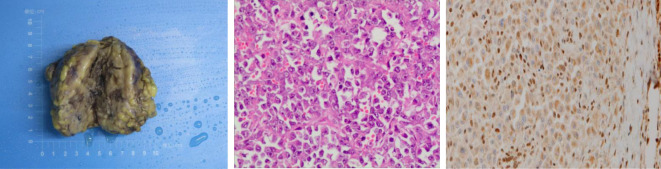
Left: Gross appearance of a local resection specimen showing a tumor with a grey-white cut surface. Middle: Microscopic appearance. Tumour is composed of epithelioid cells. H and E, x 400. Right: Loss of INI-1 expression in nuclear of tumor cells.

Based on the results from ultrasonography combined with fetal heart electronic monitoring and clinical evaluation (cervical ripening, pregnant women’s weight, and uterine height), experts in obstetrics and neonatology suggested that there was a high possibility of fetal maturity although amniocentesis was not conducted. Prophylactic corticosteroids for facilitation of fetal lung maturation were not administered due to their controversy from 34 to 36 + 6 weeks of gestation. According to guidelines from the American College of Obstetricians and Gynecologists (7), corticosteroids are recommended for women from 34 to 36 + 6 weeks of gestation who may have premature birth within 1 week. Subsequently, the obstetrics team performed preterm cesarean section, and the gynecology team performed radical local resection of the lesion on the left labia majora and left inguinal lymphadenectomy at 36 weeks and 4 days of gestation. Final pathology of the specimen demonstrated a 7-cm-sized ES with the infiltration of surrounding adipose tissue, and no necrosis, hemorrhage, or venous invasion were identified. Surgical resection margins were clear and free of tumor (distance of 2 cm from biopsy lesion). There were no lymphovascular emboli, perineurial invasion, or regional lymph node metastasis. Immunohistochemically, the tumor cells were positive for EMA, SMA, IMP3, and vimentin, while negative for S100, Desmin, Myogenin, MyoD1, and CD34. INI-1 expression was lost in tumor cells (**Figure 2**). Morphologically, it is necessary to distinguish ES from other epithelioid-looking tumors such as epithelioid peripheral nerve sheath tumor, epithelioid leiomyosarcoma, epithelioid angiosarcoma, rhabdomyosarcoma, malignant melanoma, and poorly differentiated squamous cell carcinoma. The diagnosis can only be confirmed with immunohistochemical staining, and the case was sent to a pathologist, for a second opinion, who agreed with the diagnosis of ES. The final diagnosis was proximal-type vulvar ES. The female newborn weighed 2,900 g, and the Apgar score was 9/10 (at 1 min/5 min). The newborn was not incubated and subsequently discharged uneventfully.

### After surgery

Postoperatively, the patient experienced lymphatic leakage and surgical site infection of inguinal lesions, and required 1 month for the healing of the wound. Six weeks after surgery, she presented with a recurrent painless nodule on the left labia majora, and ultrasonographic imaging revealed a subcutaneous solid mass measuring 15×13×10 mm and a solid mass of subcutaneous fat layer near the midline of the lower left abdomen. She underwent excision of the lesion. Pathology was suggestive of tumor recurrence with the infiltration of surrounding adipose tissue, no venous or neural invasion, and free-tumor surgical margins. Then, she was transferred to a tumor hospital where she received adjuvant radiotherapy with a dose of 40 Gy/10 cycles, followed by 15 Gy/3 cycles with a 10×10 cm field. The patient survived with neither recurrence nor complications at a 12-month follow-up.

## Discussion

Vulvar ES is a malignant tumor of mesenchymal origin with a recurrence rate of 38%–69% (8). This tumor manifests as having a predominantly large-cell, epithelioid cytomorphology, with marked cytologic atypia and frequent occurrence of rhabdoid features microscopically (8). Vulvar ES generally occurs in women of reproductive age, and its risk factors remain unknown due to its rarity. Immunohistochemical staining is useful for microscopic diagnosis of vulvar ES, in which a lack of staining for INI-1 protein is a characteristic finding (8). In the present case, this feature supported the pathological diagnosis of vulvar ES.

The general consensus of vulvar ES in pregnancy is immediate surgical resection (3). Currently, complete surgical excision is the best treatment option and is possible in all trimesters, but it is preferably carried out in the second or third trimester to decrease the risk of miscarriage (3). Lymphadenectomy should also be considered in the presence of lymph node metastasis (8). However, the mode of delivery and optimal opportunity for cesarean section remain experimental. In the present case, the diagnosis of vulvar ES was made in the third trimester, and ultrasonography revealed a mature fetus; thus, cesarean section in combination with tumor removal was administered. We also performed left inguinal lymphadenectomy as a staging and cytoreductive procedure, although the patient had no lymph node metastasis at the time of presentation. As she later had recurrence on the left labia majora and no groin node metastasis was observed, it may be beneficial to perform inguinal lymph node dissection in the case of an aggressive form of vulvar cancer.

Whether adjuvant therapies such as radiotherapy and chemotherapy bring favorable outcomes is unclear. Radiotherapy is commonly offered in case of positive margins or recurrent settings (8). In addition, anthracycline-based and gemcitabine-based regimens have shown some benefits in cases of unresectable or metastatic ES (9). In this case, radiotherapy is offered due to recurrence. As patients did not encounter relapse at a 12-month follow-up, it may be useful to start with radiotherapy in the case of recurrence.

It is noteworthy that vulvar ES is frequently misdiagnosed as a benign lesion such as Bartholin’s cyst, lipoma, or genital wart due to its rarity and usual presentation as a solitary painless lump (10). Thus, accurate early-stage diagnosis and management are important. Physicians should be alert when patients present with a palpable nodule in the vulva, especially in reproductive-age women, for a potentially better prognosis. Moreover, in this case, the patient and her family members were provided with the knowledge on vulvar ES with a step-by-step approach so that they were able to adjust and find strength. Through this process, the patient could accept disease-related experiences and develop a rational perspective.

## Conclusion

Aggressive approaches such as radical local resection of the lesion, cesarean section, and lymphadenectomy can improve maternal–neonatal outcomes in the case of vulvar ES during pregnancy. Moreover, radiotherapy in the case of recurrence is important.

## Data Availability

The original contributions presented in the study are included in the article/supplementary material. Further inquiries can be directed to the corresponding authors.
